# Forensic Cases in the Emergency Department: Associations Between Life-Threatening Risk, Medical Treatability, and Patient Outcomes

**DOI:** 10.3390/diagnostics15111416

**Published:** 2025-06-02

**Authors:** Harun Yildirim, Murtaza Kaya

**Affiliations:** Department of Emergency Medicine, Faculty of Medicine, Kutahya Health Sciences University, Kutahya 43100, Turkey; murtaza.kaya@ksbu.edu.tr

**Keywords:** forensic cases, emergency medicine, triage accuracy, injury severity, medico-legal documentation

## Abstract

**Background:** This study aimed to evaluate the clinical and forensic characteristics of cases admitted to a high-volume tertiary emergency department, focusing on severity-based classification using treatability with simple medical intervention (SMI) and life-threatening status. **Methods:** We retrospectively analyzed 3014 forensic cases over one year. Patients were classified based on injury severity, anatomical region, and clinical outcomes. Documentation practices and report types were also reviewed. **Results:** Among all the cases, 60.4% were treatable with SMI, and 10.5% were identified as life threatening. Notably, all patients who died (1.3% mortality) were in the life-threatening group, and none of the SMI-treated patients died, underscoring the accuracy of early triage and alignment between documentation and outcomes. Road traffic accidents were the leading cause of life-threatening injury and hospitalization, while assault cases were predominantly minor and managed conservatively. Seasonal variation peaked in July, and sex-based differences revealed a higher SMI eligibility among female patients. Final forensic reports were more frequently issued in SMI cases, while preliminary reports were predominant in severe trauma. **Conclusions:** Severity-based classification using SMI and life-threatening categories offers valuable insight for clinical decision-making and forensic documentation. Integrating structured triage, anatomical injury mapping, and standardized report templates can enhance both patient safety and legal reliability.

## 1. Introduction

Forensic cases refer to situations in which an individual’s physical or mental health is impaired due to another person’s intentional act, negligence, carelessness, or recklessness [1]. This definition encompasses the legal investigation of injuries or medical conditions and the determination of liability [2]. These include not only violent incidents such as road traffic accidents, assaults, and sexual violence, but also non-criminal assessments such as age estimation, custody-related evaluations, substance use detection, and document forgery investigations [3,4].

Emergency departments (EDs) serve as critical points for both urgent medical care and medico-legal processing [5]. Physicians in this setting are expected to provide clinical management while simultaneously fulfilling legal obligations such as injury documentation and forensic reporting to judicial authorities [6,7]. Key details—including injury type, size, location, orientation, time of occurrence, and healing timeline—must be documented thoroughly. Forensic reports are generally issued in two forms: preliminary and final. While preliminary reports are used for initial assessments, incomplete or hastily prepared versions without a detailed clinical evaluation may result in missed forensic findings and legal disadvantages for victims [8,9].

International studies highlight similar challenges. For instance, research from Saudi Arabia revealed that although 84.7% of emergency physicians were aware of the obligation to report forensic cases, 42.4% lacked adequate knowledge on how to prepare these reports properly—underscoring the need for standardized training in forensic documentation [10].

This study aims to evaluate the forensic case management and documentation practices in EDs and assess their implications on legal responsibility. By identifying deficiencies and patterns, the study seeks to support the development of improved training modules and practical guidelines for healthcare professionals.

## 2. Materials and Methods

### 2.1. Study Settings

This retrospective, descriptive, cross-sectional study was conducted in the ED of a tertiary care hospital in Turkey, which receives approximately 250,000 emergency admissions annually. The ED functions as a primary referral center and is responsible for the initial clinical and forensic assessment of cases requiring medico-legal documentation.

### 2.2. Data Source and Study Population

**Figure 1:**
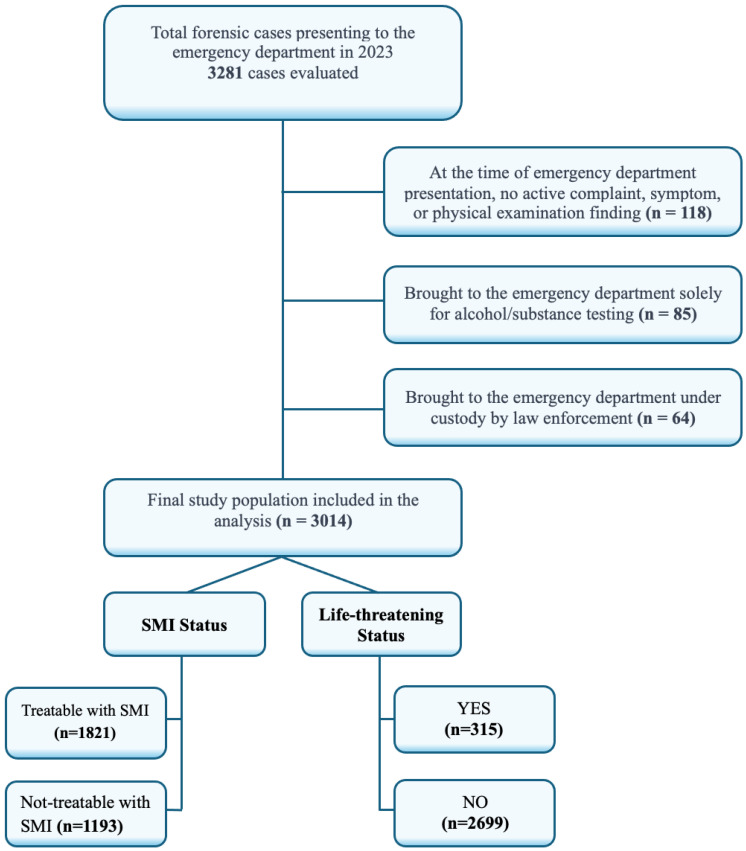
Flowchart of patient selection and inclusion criteria. SMI: simple medical intervention.

All patients classified as forensic cases who presented to the ED between 1 January 2023 and 31 December 2023 were retrospectively evaluated. Data were extracted from the hospital information management system, ED records, and official forensic report forms completed at admission. A total of 3281 patients were initially screened. After applying exclusion criteria—including non-forensic classifications, missing or inaccessible data, absence of active complaints or physical findings (*n* = 118), cases brought in solely for alcohol or substance screening (*n* = 85), and individuals admitted for custody-related procedures (*n* = 64)—a total of 3014 cases were included in the final analysis (Figure 1).

### 2.3. Variable Definitions

This study evaluated several variables including patient demographics (age, gender), seasonal trends in admissions, types of incidents resulting in forensic classification (e.g., traffic accidents, assaults), affected anatomical regions, applicability of simple medical intervention (SMI), presence of life-threatening injuries, type of forensic report issued (preliminary, final, or not issued), ED outcomes (discharge, hospitalization, referral, refusal, or death), hospital department of admission, and one-month mortality. SMI applicability and life-threatening condition were treated as independent classifications, with some non-SMI cases representing moderate but non-life-threatening injuries.

### 2.4. Statistical Analysis

All statistical analyses were performed using the Statistical Package for the Social Sciences (SPSS) software, version 27.0.1 (IBM Corp., Armonk, NY, USA). Descriptive statistics are presented as mean values and standard deviations for continuous variables, and as frequencies and percentages for categorical variables. The Shapiro–Wilk test was used to assess the normality of distribution for continuous variables. Based on the distribution characteristics, Student’s *t*-test was applied to compare the means of normally distributed continuous variables, such as age, between groups. The chi-square test was used to compare categorical variables such as gender, affected body regions, and the type of forensic report in relation to the applicability of SMI and the presence of life-threatening conditions. In cases where the expected cell counts were low, particularly in the analysis of mortality outcomes, Fisher’s exact test was employed. Binary logistic regression analysis was performed to identify independent predictors of mortality, with odds ratios (ORs) and 95% confidence intervals (CIs) reported. A *p*-value of less than 0.05 was considered statistically significant.

### 2.5. Ethical Considerations

This study was approved by the Non-Interventional Clinical Research Ethics Committee of Kütahya Health Sciences University (Approval No: 2024/02-15, dated 13 February 2024). All procedures adhered to the principles outlined in the Declaration of Helsinki. Due to the retrospective and anonymized nature of this study, the requirement for informed consent was waived.

### 2.6. Legal and Regulatory Framework

In accordance with Article 87 of the Turkish Penal Code (Law No. 5237), which came into force in June 2005, emergency physicians in Turkey are legally required to evaluate and report forensic cases using two primary criteria: (1) life-threatening condition—whether the injury poses a risk to the individual’s life, and (2) SMI—whether the injury can be treated with minimal medical care without requiring hospitalization or advanced procedures. These criteria are central to the legal classification of assault-related injuries and directly affect judicial decisions regarding the severity of the offense and corresponding penalties. To promote consistency, a national guideline titled “The Forensic Medical Assessment Guide on Injury Offenses Defined in the Turkish Penal Code” was developed by the Council of Forensic Medicine, the Turkish Association of Forensic Medicine Specialists, and the Forensic Medicine Association. The most recent version of this guide was published in June 2019 and is widely used in EDs in Turkey [11].

## 3. Results

The mean age of the patients was 32.9 ± 17.9 years. Males constituted the majority of the cases (67.3%). Seasonal distribution showed that the highest frequency of admissions occurred during the summer months (29.8%), followed by autumn (28.3%), winter (23.6%), and spring (18.3%). In terms of outcomes in the ED, 76.3% of patients were discharged, 16.5% were admitted to the hospital, 5.8% refused treatment, 1.1% were referred to other hospitals, and 0.3% died during their ED stay. Among all the cases, 60.4% were classified as treatable with SMI, while 39.6% were not. Life-threatening conditions were present in 10.5% of the cases (Table 1).

**Figure 2:**
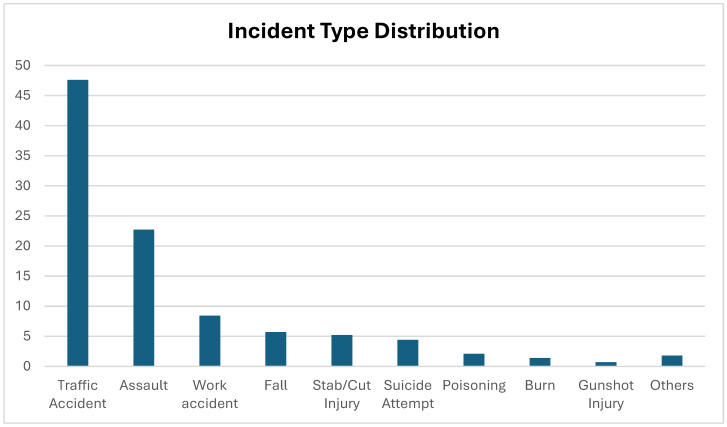
Percentage distribution of forensic cases according to incident type.

Traffic accidents were by far the most frequent cause of forensic evaluation, followed by assault and work-related injuries. This reflects the high incidence of trauma-related presentations within forensic case evaluations in emergency settings and underlines the need for efficient triage protocols and injury documentation systems (Figure 2).

Rates of SMI and life-threatening conditions varied significantly according to the type of incident. Among traffic accidents (*n* = 1436), 936 (65.2%) were treatable with SMI and 119 (8.3%) were life threatening. Assault cases (*n* = 683) also constituted a significant portion, with 80.7% being SMI-treatable and 0.9% categorized as life threatening. The third most common group was falls (*n* = 173), of which 39.3% were not treatable with SMI and 23.1% were life threatening. These three categories—traffic accidents, assaults, and falls—represented the most common reasons for forensic presentation and contributed substantially to the overall trauma burden in the ED (Table 2).

**Figure 3:**
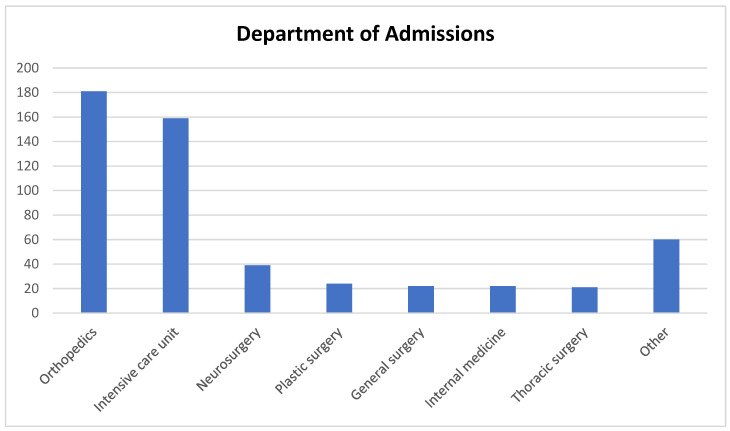
Numerical distribution of hospitalized patients across clinical departments.

Regarding clinical outcomes, 76.3% of the patients were discharged from the ED, while 16.5% required hospitalization. Among the hospitalized patients, admissions were primarily to surgical departments such as orthopedics, neurosurgery, and general surgery. Notably, 159 patients (approximately 3.4%) were admitted to the intensive care unit, reflecting the severity of certain presentations. Additionally, 1.1% of the patients were referred to other hospitals, 5.8% refused treatment, and 0.3% died during their hospital stay. These findings illustrate the diverse clinical spectrum of forensic cases and underscore the substantial burden they place on acute care resources (Figure 3).

Female patients were significantly more likely to be treated with SMI (67.5% vs. 57.0%, *p* < 0.001). Patients with multiple body system injuries were significantly more likely to require advanced treatment beyond SMI (60.6% vs. 39.4%, *p* < 0.001). This finding highlights the importance of comprehensive trauma evaluation, especially in cases involving polytrauma. Furthermore, all 39 patients who died within one month of presentation were in the non-SMI group. This supports the notion that the requirement for interventions beyond SMI is a strong predictor of poor prognosis (Table 3).

Older age was significantly associated with the presence of life-threatening injuries (mean 37.8 vs. 32.3 years, *p* < 0.001). All 39 mortality cases were in the life-threatening group (*p* < 0.001). Among patients in the life-threatening category, the most frequently affected body region was multiple body systems (*n* = 205, 65.1%). Among life-threatening cases, 95.2% (300/315) received preliminary reports, while only 1.6% (5/315) received final reports, indicating a strong association between injury severity and the preference for issuing preliminary reports (*p* < 0.001). These results underscore the value of early identification of life-threatening trauma, which is crucial for timely interventions and appropriate legal documentation (Table 3).

Binomial logistic regression analysis was conducted to identify predictors of mortality among forensic cases. The overall model was statistically significant (χ^2^(3) = 101, *p* < 0.001), with a Nagelkerke R^2^ of 0.256, indicating that approximately 25.6% of the variance in mortality was explained by the model. Among the predictors, age was found to be a significant factor (*p* < 0.001), with each additional year increasing the odds of mortality by 4% (OR = 1.04, 95% CI: 1.02–1.06). Hospital admission was also significantly associated with higher mortality risk (*p* < 0.001); admitted patients were 16.76 times more likely to die compared to those who were not hospitalized (OR = 16.76, 95% CI: 2.02–3.62). In contrast, sex was not a significant predictor of mortality (*p* = 0.117), indicating that the observed association between admission and mortality was independent of sex (Table 4).

## 4. Discussion

This study provides a comprehensive evaluation of forensic cases in a high-volume tertiary ED, highlighting the complex interplay between clinical management and judicial obligations. Emergency physicians are not only responsible for timely stabilization but also for accurate injury documentation, case classification, and legal reporting. Previous studies have shown that incomplete or inconsistent documentation may delay legal processes and weaken the judicial value of medical evidence [2,12].

Numerous studies have consistently reported a male predominance among forensic emergency cases, often concentrated in the young adult age group. For instance, Siddappa et al. noted that 71.8% of cases were male, with the highest incidence in the 21–30 age range (37.7%) [13]. Similarly, Malik et al. found that 81% of patients were male, with nearly three-quarters under 30 years of age [14]. These findings suggest that young, economically active males are more vulnerable due to increased exposure to traffic, hazardous work environments, and interpersonal violence. Our findings were consistent with this pattern: 67.3% of our patients were male, with a mean age of 32.9 years, underscoring the heightened medico-legal risk among this demographic.

The type of forensic cases presenting to EDs varies across geographic and socioeconomic contexts. Jadoon et al. identified traffic accidents (38.3%) and blunt trauma (27.3%) as leading causes in Pakistan [15], whereas Shreedhar et al. reported poisoning (40%) and traffic accidents (31.1%) as the most common in India [16]. In Turkey, Bıçakçı et al. found traffic accidents (33.3%), assault (24.1%), and occupational injuries (21.5%) to be the most prevalent [4]. In our study, traffic accidents constituted nearly half of all cases (47.7%), followed by assaults (22.7%) and work-related injuries (8.4%), which aligns more closely with Bıçakçı’s findings. This distribution may be influenced by urban density, transportation habits, industrial safety standards, and local legal reporting practices.

Seasonal trends have also been reported in forensic admissions, with several studies identifying increased case volumes in the summer months. Aydın et al. documented that 29.9% of forensic cases presented during summer [17], while Kapçı et al. similarly highlighted July, August, and September as peak months [18]. These seasonal peaks may be associated with higher mobility, outdoor activity, and increased interpersonal interactions during warmer weather. Our data mirrored this trend, with summer accounting for 29.8% of all forensic ED admissions. Recognizing this seasonal concentration may assist in resource allocation, such as augmenting staff and ensuring sufficient trauma capacity during high-incidence months.

The treatability of injuries with SMI varies by injury mechanism and anatomical involvement. Timsinha et al. reported that while most injuries involved extremities and were considered simple, the mortality rate still exceeded 8% [19]. Seviner et al. reported a life-threatening condition rate of 21.1% [20]. Within our sample, life-threatening injuries were identified in 10.5% of cases, and 39.6% were not treatable with SMI. Multisystem injuries were particularly associated with high life-threatening rates (26.9%) and low SMI-suitability (39.4%). In contrast, injuries limited to the extremities, head–neck, or torso were mostly non-life-threatening and often manageable with basic medical care. These findings highlight the need for structured anatomical assessment to inform both clinical urgency and medico-legal classification.

High rates of preliminary forensic reports have been consistently documented across EDs. Yemenici et al. reported that 68.7% of forensic reports were issued as preliminary documents [8], while Eroğlu et al. observed extreme inter-hospital variability, with temporary report rates ranging from 58.5% up to 99.6%, often shaped by institutional norms and physician habits [21]. Akbaba et al. further noted that temporary reports were frequently issued even in high-impact injuries such as traffic accidents and penetrating trauma, frequently without clear medical or legal justification—highlighting the disconnect between emergency physicians’ assessments and those of forensic medicine specialists [22]. Consistent with these findings, preliminary reports were issued in 67.3% of cases, while only 32.7% received definitive forensic documentation. This pattern not only mirrors previous findings but also underscores an ongoing reliance on provisional evaluations in emergency settings. Such practices may stem from clinical workload, medico-legal uncertainty, or lack of forensic training, but they carry significant risks—delaying judicial proceedings, weakening the evidentiary value of medical documentation, and potentially undermining victims’ access to justice.

Hospital admission rates among forensic cases reported in the literature vary considerably, often depending on the severity spectrum of included injuries. Aslaner et al. reported a hospitalization rate of 6.6% among forensic cases presenting to a secondary care hospital, with even lower rates (2.6%) among those with repeated ED visits, largely attributed to minor injuries such as interpersonal violence [23]. Similarly, Yüzbaşıoğlu et al. found a 5.8% admission rate in a refugee population, with most cases managed on an outpatient basis [24]. These lower rates likely reflect cohorts dominated by less severe presentations or barriers to care such as socioeconomic or legal status. In contrast, our study reported a hospital admission rate of 16.5%, indicating a comparatively higher injury burden. This discrepancy may reflect the inclusion of a broader range of high-energy injuries—particularly falls, suicide attempts, and occupational trauma—often requiring multidisciplinary inpatient care. Most admitted patients in our cohort were referred to surgery, orthopedics, or neurosurgery, highlighting the need for specialized trauma care in forensic cases with significant physiological impact.

Mortality rates reported in forensic emergency populations have shown considerable heterogeneity across studies and settings. In a tertiary hospital in North India, Mir et al. reported an in-hospital mortality rate of 11.7% among medico-legal cases, with a high proportion of severe trauma and delayed presentations likely contributing to this figure [25]. Kumar et al. documented a mortality rate of 4.15%, with road traffic accidents and blunt force injuries identified as the primary contributors [26]. These elevated rates may reflect limitations in prehospital care access, delayed intervention, or higher overall trauma acuity in those settings. In our cohort, the 1-month mortality rate was 1.3%, which is comparatively lower. Fatalities were mostly attributed to high-energy mechanisms, including traffic accidents (*n* = 18), falls (*n* = 9), suicide attempts (*n* = 2), and work-related injuries (*n* = 5). Notably, there were no deaths following stab/cut wounds, gunshot injuries, or burns—possibly indicating earlier intervention or lower injury severity in these categories. Our exclusion of brought-in-dead cases and late post-discharge deaths may also partially explain the lower mortality rate. Together, these findings emphasize the role of early access to definitive trauma care and system-level responsiveness in reducing preventable deaths in forensic populations.

## 5. Limitations

This study has several limitations that should be acknowledged. First, the retrospective design may introduce selection and information bias, as data collection was based on routine medical and forensic records, which may contain omissions or inconsistencies. Additionally, evaluator variability among emergency physicians and the time-sensitive nature of forensic documentation may have affected the completeness and consistency of the data. Second, although the sample size is large, this study reflects the experience of a single tertiary center and may not fully represent national patterns. Moreover, long-term outcomes, such as legal adjudication results or long-term functional disability, were not captured due to data access constraints. Lastly, while this study classified injury severity based on clinical presentation and disposition, no standardized scoring system was used, which may affect reproducibility across different institutions.

## 6. Conclusions

This study presents a detailed examination of the clinical and legal characteristics of forensic cases admitted to a tertiary ED. By utilizing nationally recognized medico-legal criteria—such as the presence of life-threatening injuries and the applicability of SMI—we demonstrated that case classification is not only legally relevant but also associated with important clinical outcomes, including hospitalization and mortality. The observed consistency between physicians’ assessments and legal documentation highlights the dual responsibility of emergency professionals in both clinical management and judicial accuracy. These findings emphasize the importance of improving forensic awareness and documentation practices among emergency physicians. National policies and training programs tailored to medico-legal responsibilities in emergency care may contribute to better patient outcomes and greater legal reliability.

## Figures and Tables

**Table 1:** Demographic characteristics of the patients.

Age (Mean ± SD)		32.9	±17.9
Sex *n* (%)	Male	2028	67.3
	Female	986	32.7
Seasonal Distribution *n* (%)	Spring	553	18.3
	Summer	897	29.8
	Autumn	853	28.3
	Winter	711	23.6
Outcome in ED *n* (%)	Discharged	2299	76.3
	Admitted to Hospital	498	16.5
	Refused the Treatment	174	5.8
	Referral to Another Hospital	34	1.1
	Died in ED	9	0.3
Life-Threatening Condition?	Yes	315	10.5
	No	2699	89.5
Simple Medical Intervention	Treatable	1821	60.4
	Not Treatable	1193	39.6

ED: emergency department; Spring: March, April, May; Summer: June, July, August; Autumn: September, October, November; Winter: December, January, February.

**Table 2:** Distribution of forensic cases by incident type: demographics, clinical severity, and outcomes.

	SEX		AGE	SMI		LTC		H	MO
*n* (%)	M 2028 (67.3)	F 964 (32.7)	Mean	Yes 1821 (60.4)	No 1193 (39.6)	Yes 315 (10.5)	No 2699 (89.5)	(+) 498 (16.5)	(+) 39 (1.3)
Traffic Accident	964 (32.0)	472 (15.7)	33.1	936 (31.1)	500 (16.6)	119 (3.9)	1317 (43.7)	245 (8.1)	18 (0.6)
Assault	427 (14.2)	256 (8.5)	32.3	551 (18.3)	132 (4.4)	6 (0.2)	677 (22.5)	20 (0.7)	0 (0)
Work Accident	220 (7.3)	33 (1.1)	35.7	114 (3.8)	139 (4.6)	19 (0.6)	234 (7.8)	46 (1.5)	5 (0.2)
Fall	117 (3.9)	56 (1.8)	33.5	68 (2.3)	105 (3.5)	40 (1.3)	133 (4.4)	65 (2.2)	9 (0.3)
Stab/Cut Injury	131 (4.3)	27 (0.9)	28.5	51 (1.7)	107 (3.6)	27 (0.9)	131 (4.3)	36 (1.2)	0 (0)
Poisoning	27 (0.9)	35 (1.2)	38.5	42 (1.4)	20 (0.7)	11 (0.4)	51 (1.7)	7 (0.2)	0 (0)
Suicide Attempt	62 (2.0)	71 (2.4)	31.0	19 (0.6)	114 (3.8)	67 (2.2)	66 (2.2)	54 (1.8)	2 (0.1)
Burn	22 (0.7)	19 (0.6)	20.7	15 (0.5)	26 (0.9)	9 (0.3)	32 (1.1)	9 (0.3)	0 (0)
Substance/Alcohol	18 (0.6)	4 (0.1)	33.5	10 (0.3)	12 (0.4)	6 (0.2)	16 (0.5)	4 (0.1)	2 (0.1)
Electrical Injury	9 (0.3)	4 (0.1)	34.5	6 (0.2)	7 (0.2)	2 (0.1)	11 (0.4)	0 (0)	0 (0)
Sexual Assault	2 (0.1)	3 (0.1)	24.8	3 (0.1)	2 (0.1)	0 (0)	5 (0.2)	0 (0)	0 (0)
Animal Attack	6 (0.2)	3 (0.1)	41.7	3 (0.1)	6 (0.2)	1 (0.0)	8 (0.3)	3 (0.1)	0 (0)
Drowning	3 (0.1)	1 (0)	33.3	2 (0.1)	2 (0.1)	2 (0.1)	2 (0.1)	1 (0)	1 (0)
Gunshot Injury	18 (0.6)	2 (0.1)	39.0	1 (0)	19 (0.6)	4 (0.1)	16 (0.5)	8 (0.3)	0 (0)
Suspicious Death	2 (0.1)	0 (0)	71.0	0 (0)	2 (0.1)	2 (0.1)	0 (0)	0 (0)	2 (0.1)

SMI: simple medical intervention; LTC: life-threatening condition; H: hospitalized; MO: mortality; M: male; F: female.

**Table 3:** Comparison of forensic case characteristics by SMI eligibility and life-threatening risk.

*n* (%)		Treatable with SMI 1821 (60.4)	Not Treatable with SMI 1193 (39.6)	*p*-Value	Life Threatening: YES 315 (10.5)	Life Threatening: NO 2699 (89.5)	*p*-Value
Sex	Male	1155 (57.0)	873 (43.0)	<0.001 ^a^	214 (10.6)	1814 (89.4)	0.795 ^a^
	Female	666 (67.5)	320 (32.5)		101 (10.2)	885 (89.8)	
Age		31.2 ± 17.3	35.4 ± 18.4	<0.001 ^b^	37.8 ± 20.0	32.3 ± 17.5	<0.001 ^b^
Mortality	Yes	0 (0)	39 (100)	<0.001 ^c^	39 (100)	0 (0)	<0.001 ^c^
	No	1821 (61.2)	1154 (38.8)		276 (9.3)	2699 (90.7)	
Affected Body Region							
Head, Neck, and Face		561 (71.4)	225 (28.6)	<0.001 ^a^	48 (6.1)	738 (93.9)	<0.001 ^a^
Chest and Back		157 (66.2)	80 (33.8)		21 (8.9)	216 (91.1)	
Abdomen, Pelvic, and Genital Regions		97 (53.6)	84 (46.4)		24 (13.3)	157 (86.7)	
Upper and/or Lower Extremities		706 (67.3)	343 (37.2)		17 (1.6)	1032 (98.4)	
Multiple Body Systems		300 (39.4)	461 (60.6)		205 (26.9)	556 (73.1)	
Forensic Report Type							
Preliminary Report		574 (32.9)	1171 (67.1)	<0.001 ^a^	300 (17.2)	1445 (82.8)	<0.001 ^a^
Final Report		1242 (99.6)	5 (0.4)		5 (0.4)	1242 (99.6)	
Not Issued		5 (22.7)	17 (77.3)		10 (45.5)	12 (54.5)	

^a^: chi-square test, ^b^: Student’s *t*-test, ^c^: Fisher’s exact test, SMI: simple medical intervention.

**Table 4:** Logistic regression results for mortality. Model coefficients—mortality.

Predictor	Estimate	Lower CI	Upper CI	*p*-Value	Odds Ratio
Intercept	−7.524	−8.59	−6.45	<0.001	5.40
Age	0.039	0.02	0.05	<0.001	1.04
Hospitalized	2.819	2.02	3.61	<0.001	16.76
Sex	0.540	−0.13	1.21	0.117	1.72

## Data Availability

The datasets used and/or analyzed during the current study are available from the corresponding author on reasonable request. The data are not publicly available due to privacy.

## Abbreviations

SMI	Simple medical intervention
ED	Emergency department
LTC	Life-threatening condition
H	Hospitalized
MO	Mortality
M	Male
F	Female
